# Complete chloroplast genome of *adonis amurensis* (ranunculaceae), an important cardiac folk medicinal plant in east asia

**DOI:** 10.1080/23802359.2021.1875916

**Published:** 2021-02-12

**Authors:** Li-Qiu Zhang, Xiao-Yan Zhang, Yan-Wu Hu, Ren-Shuang Sun, Jun-Lin Yu

**Affiliations:** a School of Medicine and Pharmacy, Tonghua Normal University, Tonghua, China; b School of food science and Engineering, Tonghua Normal University, Tonghua, China

**Keywords:** *Adonis amurensis*, Ranunculaceae, chloroplast genome, phylogenomics

## Abstract

*Adonis amurensis* Regel et Radde is an important cardiac folk medicinal plant which endemic to Northeast Asia. We determined the first complete chloroplast genome of *A. amurensis* using genome skimming approach. The cp genome was 157,032 bp long, with a large single-copy region (LSC) of 86,218 bp and a small single-copy region (SSC) of 18,212 bp separated by a pair of inverted repeats (IRs) of 26,301 bp. It encodes 129 genes, including 84 protein-coding genes, 37 tRNA genes, and 8 ribosomal RNA genes. We also reconstructed the phylogeny of Adonideae and Isopyreae using maximum likelihood (ML) method, including our data and previously reported cp genomes of related taxa. The phylogenetic analysis indicated that *A. amurensis* is close related with *Adonis sutchuenensis*.

The genus *Adonis* L. (Ranunculaceae), native to Europe and Asia, comprises 32 annual or perennial herbaceous species. Due to their cardiac-enhancing effects, *Adonis* spp. have long been used in European and Chinese folk medicine (Shang et al. 2019). *Adonis amurensis* Regel et Radde is morphologically distinguished from other taxa of *Adonis* by unbranched glabrous stem, long leaf petioles branched two or three times, one flower with eight to nine sepals, and sepals longer to that of petals (Gorovoy and Gurzenkov 1969; Wang 1980; Nishikawa and Kadota 2006; Son 2015). However, because of the morphological characters that are shared among objective distinctive characteristics of the taxa within the genus and the diverse morphological variation of *A. amurensis*, it has been difficult to identify taxa and investigate their phylogenetic relationships, and hence taxonomic status of *A. amurensis* has been treated in very contrasting ways (Son 2015; Son et al. 2018). Despite recent studies on the genus *Adonis* from East Asia (Kaneko et al. 2008; Son et al. 2016; Son et al. 2017), the taxonomic and phylogenetic relationships among taxa still need to be confirmed by more molecular evidences. By taking advantages of next-generation sequencing technologies that efficiently provide the chloroplast (cp) genomic resources of our interested species, we can rapidly access the abundant genetic information for phylogenetic research and conservation genetics (Li et al. 2017; Liu et al. 2017). Therefore, we sequenced the whole chloroplast genome of *A. amurensis* to elucidate its phylogenetic relationship within Ranunculaceae.

Total genomic DNA was extracted from silica-dried leaves collected from the campus of Tonghua Normal University using a modified CTAB method (Doyle and Doyle 1987). The voucher specimen (sfxyyyxycjzh20200429) was collected and deposited in the Herbarium of Tonghua Normal University (41°44′40.69″, 125°58′57.63″, 427.3). DNA libraries preparation and pair-end reads sequencing were performed on the Illumina NovaSeq 6000 platform. The cp genome was assembled via NOVOPlasty (Dierckxsens et al. 2017), using the *A. sutchuenensis* cp genome (MK569470, Zhai et al. 2019) as a reference. Gene annotation was performed via the online program Dual Organellar Genome Annotator (DOGMA; Wyman et al. 2004). Geneious R11 (Biomatters Ltd., Auckland, New Zealand) was used for inspecting the cp genome structure.

The complete cp genome of *A. amurensis* (GenBank accession MW042677) was 157,032 bp long consisting of a pair of inverted repeat regions (IRs with 26,301 bp) divided by two single-copy regions (LSC with 86,218 bp; SSC with 18,212 bp). The overall GC contents of the total length, LSC, SSC, and IR regions were 38.0%, 36.2%, 31.5% and 43.1%, respectively. The genome contained a total of 129 genes, including 84 protein-coding genes, 37 tRNA genes and 8 rRNA genes.

We used a total of 15 additional complete cp genomes of the Ranunculaceae species to clarify the phylogenetic position of *A. amurensis*. *Asteropyrum cavaleriei* (NC041530), *Callianthemum taipaicum* (NC041476) and *Anemonopsis macrophylla* (NC041527) were used as the outgroup. We reconstructed a phylogeny employing the GTR + G model and 1000 bootstrap replicates under the maximum-likelihood (ML) inference in RAxML-HPC v.8.2.10 on the CIPRES cluster (Miller et al. 2010). The ML tree (Figure 1) was consistent with the most recent phylogenetic study on Ranunculaceae (Son et al. 2016; Zhai et al. 2019). *A. amurensis* exhibited the closest relationship with *Adonis sutchuenensis.*

**Figure 1.**
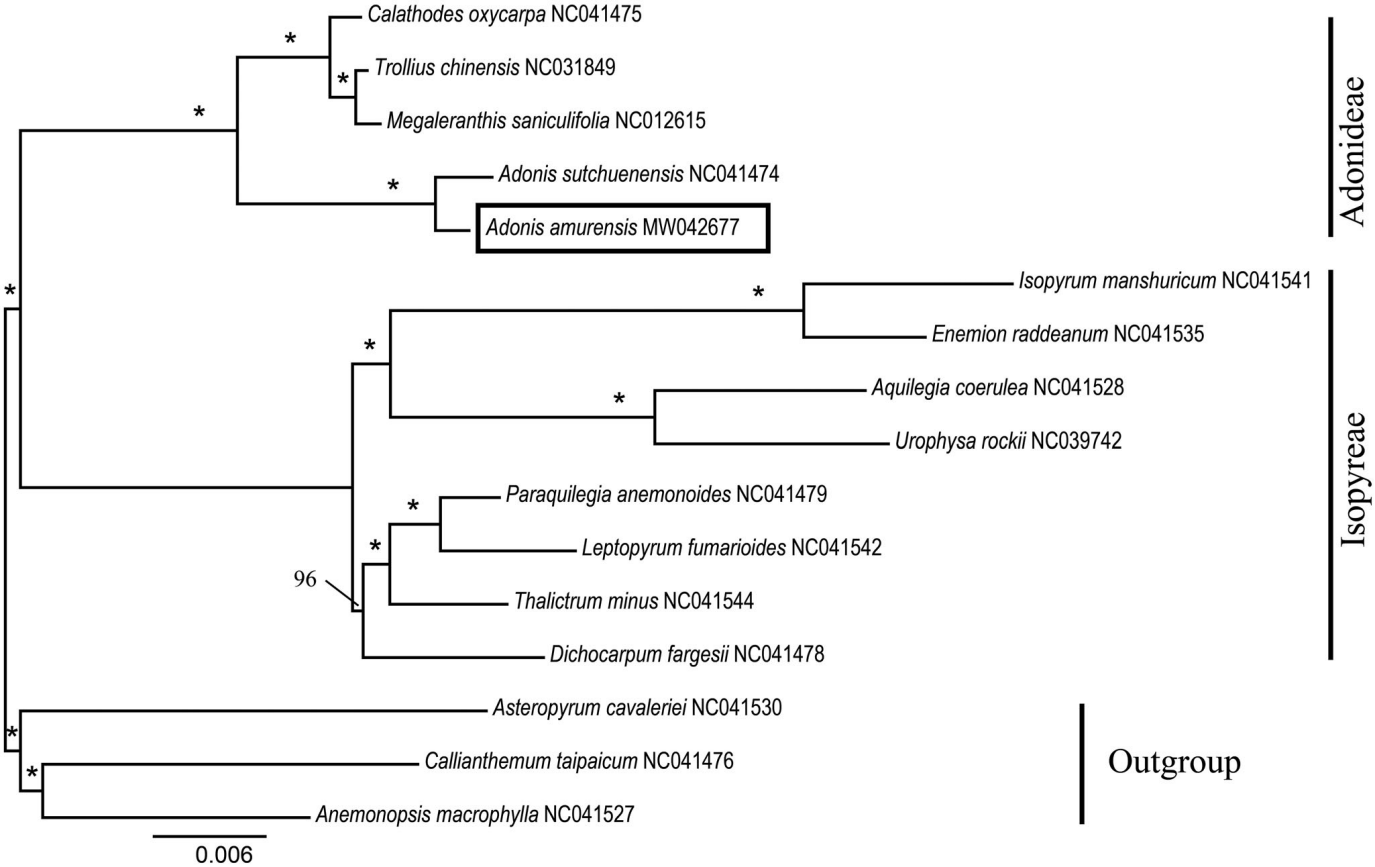
Phylogenetic tree reconstruction of 16 taxa of Ranunculaceae using ML method. Relative branch lengths are indicated. Support values above the branches are ML bootstrap support; “*” indicates 100% support values.

## Data Availability

The data that support the findings of this study are openly available in GenBank of NCBI at https://www.ncbi.nlm.nih.gov, reference number MW042677.
